# 3D Bioprinting of an In Vitro Model of a Biomimetic Urinary Bladder with a Contract-Release System

**DOI:** 10.3390/mi13020277

**Published:** 2022-02-09

**Authors:** Suhun Chae, Jaewook Kim, Hee-Gyeong Yi, Dong-Woo Cho

**Affiliations:** 1 Department of Mechanical Engineering, Pohang University of Science and Technology (POSTECH), Pohang 37673, Korea; csh1528@postech.ac.kr (S.C.); ki9204@postech.ac.kr (J.K.); 2 Department of Rural and Biosystems Engineering, College of Agriculture and Life Sciences, Chonnam National University, Gwangju 61186, Korea; 3 Institute for Convergence Research and Education in Advanced Technology, Yonsei University, Seoul 03722, Korea

**Keywords:** in vitro bladder model, 3D bioprinting, decellularized bladder extracellular matrix, bladder tissue engineering

## Abstract

The development of curative therapy for bladder dysfunction is usually hampered owing to the lack of reliable ex vivo human models that can mimic the complexity of the human bladder. To overcome this issue, 3D in vitro model systems offering unique opportunities to engineer realistic human tissues/organs have been developed. However, existing in vitro models still cannot entirely reflect the key structural and physiological characteristics of the native human bladder. In this study, we propose an in vitro model of the urinary bladder that can create 3D biomimetic tissue structures and dynamic microenvironments to replicate the smooth muscle functions of an actual human urinary bladder. In other words, the proposed biomimetic model system, developed using a 3D bioprinting approach, can recreate the physiological motion of the urinary bladder by incorporating decellularized extracellular matrix from the bladder tissue and introducing cyclic mechanical stimuli. The results showed that the developed bladder tissue models exhibited high cell viability and proliferation rate and promoted myogenic differentiation potential given dynamic mechanical cues. We envision the developed in vitro bladder mimicry model can serve as a research platform for fundamental studies on human disease modeling and pharmaceutical testing.

## 1. Introduction

The primary function of the urinary bladder is to store and expel urine by optimizing the intravesical pressure regulated by the detrusor muscle in the bladder wall [1]. In particular, the smooth muscle cells of the bladder play an essential role in maintaining bladder compliance [2]. Abnormal bladder compliance, such as pathological conditions of the bladder wall, due to abnormal and uncontrollable detrusor contractions leading to slow or incomplete bladder emptying, can cause urinary tract infection, urinary incontinence, and renal failure [3,4]. Unfortunately, precise diagnosis and treating of dysfunctions accurately is still a clinical challenge [5]. This can be overcome using a tissue engineering approach, which provides a potential solution for producing a tissue model of the human bladder to realize in vivo bladder functions. However, current in vitro models cannot recreate all structural and biophysical environments of the bladder [6]. Therefore, it is necessary to develop a reliable model system that closely mimics the physiological and biological features of actual human bladder tissue to support pathological studies pertaining to bladder diseases.

Compared to conventional cell culture or experimental animal models, three-dimensional (3D) in vitro models, such as organ-on-a-chip, are an emerging technology that replicates the key properties of structures, functions, and biological conditions of human tissues/organs in a miniaturized platform by introducing relevant cell populations with tissue/organ-specific environmental and biomechanical cues [7,8,9]. Developing functional units of human tissues/organs in vitro can facilitate translational applications, such as human healthy/disease modeling, drug screening, and precision medicine [7,10]. To date, several in vitro microphysiological systems have been developed to model the human brain, heart, lungs, kidneys, and skin, and have gained immense popularity in tissue development and disease modeling studies, and in testing therapeutic drugs [8,11,12,13,14]. However, none of the models could create a complex biomimetic bladder model that provides specific structural and mechanical environments in a functional micro-tissue unit.

In parallel, biofabrication techniques have been developed to produce 3D cellular structures in tissue/organ model systems [15]. 3D bioprinting technology is a powerful tool for engineering in vitro models [16,17]. Recently, several attempts have been made to emulate physiologically relevant microenvironments by engaging the spatial organization capability of bioprinting techniques to increase the complexity of the construct and improve its similarity to native tissues [18]. Cell-laden hydrogel, namely bioink, is an essential element in 3D bioprinting and provides a suitable microenvironment for the cells [19]. A number of biomaterials, such as fibrin, alginate, and collagen, are widely used as bioink formulations [20,21]. However, single component-based materials lack biological complexity and cannot fully recapitulate native tissue microenvironments [18,19,22]. Conversely, decellularized extracellular matrix (dECM) can recreate a tissue-specific milieu with various ECM components, including collagen, glycosaminoglycans (GAGs), laminin, fibronectin, and glycoproteins. Furthermore, considering each tissue exhibits unique components and organization of cells and ECM, it is crucial to provide intricate microenvironments with cells to achieve functional similarity to the actual tissue/organ [19,22]. Recent studies have demonstrated the use of bladder-derived dECM material as cellular scaffolds with the ability to improve the capacity of in vitro myogenic induction, and to promote in vivo bladder regeneration in a rat model after implantation, suggesting the potential of using bladder tissue-specific dECM for tissue engineering applications [23,24]. Therefore, a combinational approach of 3D bioprinting using tissue-specific bioink can be useful in engineering 3D in vitro model systems with high complexity and biofunctionality.

**Figure 1:**
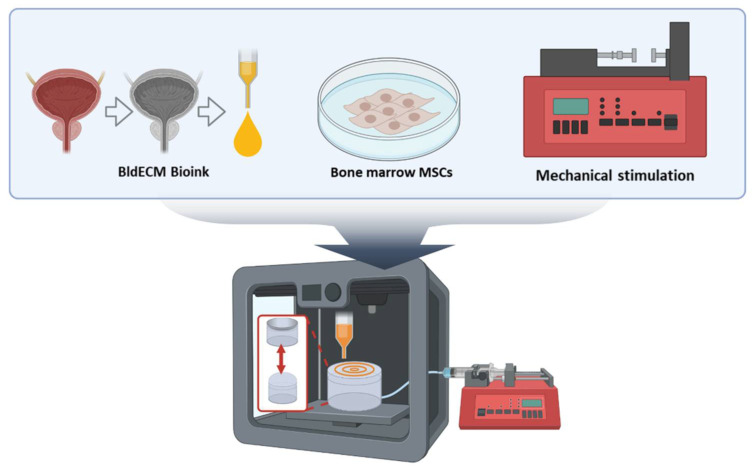
A schematic of the development of the in vitro bladder model system using 3D bioprinting and tissue-specific bioink in conjunction with the physiological stimulation system.

In this study, we propose an advanced in vitro bladder model using bladder-specific dECM bioink and 3D bioprinting technology to emulate the smooth muscle functions of the urinary bladder. We hypothesize that incorporating native bladder-like ECM with physiological mechanical stimuli can improve the biofunctionality of the 3D bladder tissue model (Figure 1). To evaluate this, we developed an in vitro bladder smooth muscle tissue model using 3D bioprinting with bladder-specific dECM bioink. Additionally, we used the developed in vitro model to investigate the influence of mechanical stimuli on myogenic differentiation by establishing a biomimetic bladder model to corroborate the synergistic effects of the surrounding matrices and physiological stimulation on the maturation of the bladder smooth muscle cells. We believe that the manufacturing design and process can be used to develop biomimetic experimental platforms for bladder tissue engineering applications.

## 2. Materials and Methods

### 2.1. Preparation of the dECM Bioink

#### 2.1.1. Decellularization Protocol of Bladder Tissue-Derived dECM (BldECM)

The decellularization protocol of the bladder was developed in reference to a previous method with some modifications [23,24]. Briefly, porcine bladder tissue was harvested from a nearby slaughterhouse with the supplier’s approval. Urothelium and lamina propria were mechanically delaminated from the native bladder to collect the muscle layers of the bladder. The muscular tissues of the bladder were then chopped into small slices with a thickness of 1 mm to increase the surface area and thereby reduce the decellularization time. After rinsing with distilled water for 1 h, the chopped tissues were treated with 1% (*w*/*v*) sodium dodecyl sulfate (SDS; BioShop Burlington, Canada) in phosphate-buffered saline (PBS; T&I, Chuncheon, Korea) solution for 72 h, and then treated with 1% (*v*/*v*) TritonX-100 (Biosesang, Seongnam, Korea) in PBS solution for 12 h. Following the detergent treatment steps, the tissues were stirred with isopropanol (Samchun, Pyeongtaek, Korea) for 12 h to remove any residual lipids. The decellularized tissues were treated using a disinfecting solution comprising 0.1% (*v*/*v*) peracetic acid (Sigma-Aldrich, Saint Louis, MO, USA) and 4% (*v*/*v*) ethanol for 4 h. After each step, the tissues were thoroughly rinsed with PBS solution to remove residual agents. Finally, the decellularized bladder tissues were lyophilized and stored at −20 °C until use.

#### 2.1.2. Biochemical Assay of BldECM

The efficacy of the decellularization method was evaluated through biochemical assays. The residual DNA was measured using a DNA extraction kit (GeneJET Genomic DNA Purification Kit, Thermo Scientific, Rockford, IL, USA) to isolate the DNA elements from the native and decellularized bladder tissues and quantify the residual double-strand DNA concentration after decellularization. Furthermore, the collagen contents in the native bladder and BldECM were evaluated using a hydroxyproline assay (Hydroxyproline Assay Kit, Sigma-Aldrich). The GAG contents were validated using 1,9-dimethylmethylene blue (Sigma-Aldrich) solution and measured at 492 nm using a microplate reader (Epoch 2 Microplate Spectrophotometer, BioTek, Winooski, VT, USA). All experiments were conducted as per their manufacturers’ protocol.

#### 2.1.3. Preparation of BldECM Bioink

The lyophilized decellularized tissues were ground to obtain fine BldECM powders, which were then enzymatically digested with a solution of pepsin (2 mg/mL; Sigma-Aldrich) in 0.5 M acetic acid (Merck Millipore, Billerica, MA, USA) to achieve a final concentration of 20 mg/mL. For comparison, we used type I atelocollagen (collagen; Dalim Tissen, Seoul, Korea) dissolved in an acetic acid solution with pepsin at a final concentration of 20 mg/mL. After solubilization for 4–5 days, the acidic pre-gel solution was neutralized to pH 7.4 by adding 10 M sodium hydroxide (Biosesang) dropwise on ice to avoid gelation. The pH-adjusted pre-gel solution was stored at 4 °C until use.

### 2.2. Cell Isolation and Culture

Human bone marrow-derived mesenchymal stem cells (hBMSCs; Catholic MASTER Cells, passage 2) were obtained from the iliac crest of a young healthy male donor supplied by the Catholic Institute of Cell Therapy (CIC; Seoul, Korea; www.cic.re.kr (accessed on 8 February 2022)) with approval from the Institutional Review Board of Seoul St. Mary’s Hospital, The Catholic University of Korea (PIRB-2018-E083). The isolated cells were cultured and expanded in growth medium with low glucose DMEM (Hyclone, Logan, UT, USA) supplemented with 20% fetal bovine serum (FBS; Hyclone) and 1% penicillin–streptomycin (P/S; Hyclone) at 37 °C in 5% CO_2_. The growth medium was replaced every 2 days until the cells reached 80–90% confluence. Cells used in all experiments were up to passage 5.

### 2.3. Fabrication of the Acrylic-Based Housing Platform

The self-designed acrylic housing platform was fabricated using a laser cutter and compartmentalized into a baseplate and a wall template with dimensions of 50 mm × 50 mm × 20 mm (length × width × height) to serve as a vacuum chamber and to store the culture medium, respectively.

A polydimethylsiloxane (PDMS; Dow Corning, Midland, TX, USA) solution was prepared by mixing the elastomer with a curing agent in a 10:1 ratio. The PDMA solution was spin-coated on the silicon wafer at 1000 rpm for 30 s and subsequently cured in the oven at 80 °C for 2 h to obtain a PDMS membrane with a thickness of 90–130 μm. Then, the PDMS membrane was cut into smaller units of 40 mm × 40 mm (length × width) to be used as bioprinting substrates.

To set up an integrated housing platform, the PDMS membrane was placed above the baseplate, and the wall template was firmly attached to it and assembled using a hot melt adhesive glue gun. Next, we conducted oxygen plasma treatment on a PDMS membrane at a power of 100 W for 5 min and an oxygen flow rate of 100 sccm using the CUTE plasma system (Femto Science Inc., Hwaseong, Korea) to achieve a hydrophilic surface. All PDMS membranes were gently washed with ethanol and sterilized using UV light. The final platforms were used for 3D bioprinting of the bladder models.

### 2.4. 3D Bioprinting Process of the In vitro Bladder Model

The in vitro bladder models were printed using a custom-built 3D bioprinter, an integrated composite tissue/organ building system (ICBS) [25]. The hBMSCs were homogeneously mixed with a 2% (*w*/*v*) BldECM pre-gel solution (cell density: 5 × 10^6^/mL) to prepare cell-laden BldECM bioink. The bioink was then loaded into a sterilized 10 mL plastic syringe with a nozzle diameter of 300 μm and dispensed onto the PDMS membrane. Throughout the bioprinting process, the temperature of the print head was maintained at 10 °C to avoid bioink gelation. According to the programmed G-code, various constructs with pre-designed patterns, including parallel, contour, and cross types, were created using a pneumatic pressure of 65 kPa and a printing head speed of 80 mm/min. To induce the cross-linking, all bioprinted constructs were immediately incubated at 37 °C for 30 min. Subsequently, the cross-linked bladder constructs were immersed in culture medium. The cell-laden constructs of a parallel-type structure were used in all in vitro experiments.

### 2.5. Establishing a Contract-Release System for Physiological Stimulation

To provide physiological stimuli and mimic the contractile motion of the bladder smooth muscle, a contract-release system (CRS) was developed using an integrated housing platform. The syringe pump (KD Scientific, Holliston, MA, USA), medical syringes, and silicon tubes (ID 0.5 mm) were sterilized and exploited to construct the CRS. One end of a silicone tube was connected to a 10-mL medical syringe, whereas the other end was inserted into the baseplate of the acrylic housing platform. For mechanical stretching, we applied 5% cyclic strain at a frequency of 0.1 Hz in a pulsatile manner for 10 d, according to previous reports [26,27]. By controlling the direction of airflow generated and manipulating it through a programmable syringe pump, the structure of the PDMS membrane was periodically altered to reflect the physiologic stretching of the bladder smooth muscle. This cyclic stretch was continued for 16 h daily and the constructs remained at rest for 8 h (to replicate normal bladder activity during the night). The entire model was immersed in culture medium during stimulation for 10 d.

### 2.6. Live/Dead Assays

The viability of the hBMSCs in the BldECM bioink was evaluated using a live/dead viability kit (LIVE/DEAD Viability/Cytotoxicity Kit, Invitrogen, Waltham, MA, USA) according to the manufacturer’s protocol. After culturing for 1, 5, and 10 d in vitro, the cell-printed BldECM constructs were washed with PBS three times. Subsequently, the samples were incubated in a staining solution comprising calcein AM and ethidium homodimer at 37 °C for 1 h. Then, the stained samples were visualized using a confocal laser scanning microscope. The green and red fluorescence represented the live and dead cells, respectively.

### 2.7. Cell Proliferation Rate

The proliferation rate of the cell-laden BldECM construct was evaluated using the Cell Counting Kit-8 (CCK-8 kit, Dojindo Molecular Technologies, Kumamoto, Japan) and DNA extraction kit as per the manufacturers’ protocol. For the CCK-8 test, the cell-laden BldECM constructs were washed with PBS solution at predetermined time points (1 d, 5 d, and 10 d) and incubated in a CCK-8 kit solution containing culture medium. Cell-laden collagen constructs were used for comparison. At each time point, the absorbance of both groups was measured at 450 nm and compared. For DNA quantification, the samples were quantified using a DNA extraction kit as per the manufacturer’s protocol. The dsDNA concentration was measured with a NanoDrop instrument (Thermo Fisher Scientific).

### 2.8. Real-Time Polymerase Chain Reactions

A quantitative real-time polymerase chain reaction (qRT-PCR) test was performed to validate the smooth muscle cell differentiation from the hBMSCs-laden bladder models, either statically or dynamically. Briefly, the total RNA was isolated from the samples at each time point using TRIZOL (Invitrogen), and the concentrations and purity of the extracted RNA were determined using a NanoDrop spectrometer. For the qRT–PCR of mRNA transcripts, first-strand complementary DNAs (cDNAs) were synthesized using a cDNA synthesis kit (Thermo Fisher Scientific). Furthermore, the transcriptional level of the target genes was examined with SYBR-green using a StepOne Plus RealTime PCR System (Applied Biosystem, Austin, TX, USA). The housekeeping gene of glyceraldehyde 3-phosphate dehydrogenase (GAPDH) was used to normalize the relative gene expression and was analyzed using the 2^−ΔΔCT^ method. The primer sequences are listed in Table 1. All procedures were performed according to the manufacturer’s protocols.

### 2.9. Immunofluorescence Staining

Immunofluorescence staining was conducted to determine the expression of smooth muscle markers. At predetermined time periods, the cultured samples were fixed in 4% paraformaldehyde (Chembio, Hanam, Korea) for 30 min. After washing with PBS, the samples were permeabilized with a 0.1% Triton X-100 solution for 30 min and blocked with 1% bovine serum albumin (BSA) in PBS for 30 min. Subsequently, the samples were incubated with anti-α-SMA (Abcam) diluted in a blocking buffer (1:200; antibody to 1% BSA in PBS) overnight at 4 °C, followed by incubation using Alexa Fluor^®^ 594 goat anti-mouse antibody (ThermoFisher Scientific) in PBS at 21 °C for 2 h. For staining of the actin filament, the samples were incubated with phalloidin-FITC (ThermoFisher Scientific) according to the manufacturer’s protocol. All samples were counterstained with DAPI mounting solution and the results were visualized using a confocal microscope (FluoView, FV1000, Olympus, Tokyo, Japan) with Olympus FluoView version 4.3 software.

### 2.10. Statistical Analysis

All data are represented as means ± standard deviation (SD). Statistical analysis was performed using GraphPad Prism software (version 8.0, GraphPad Software), and an unpaired Student’s *t*-test was used to compare the groups of in vitro experiments. Statistical significance was set at *p* < 0.05 (*) and *p* < 0.01 (**).

## 3. Results and Discussion

### 3.1. Preparation of the Bladder-Derived dECM Bioink

**Figure 2:**
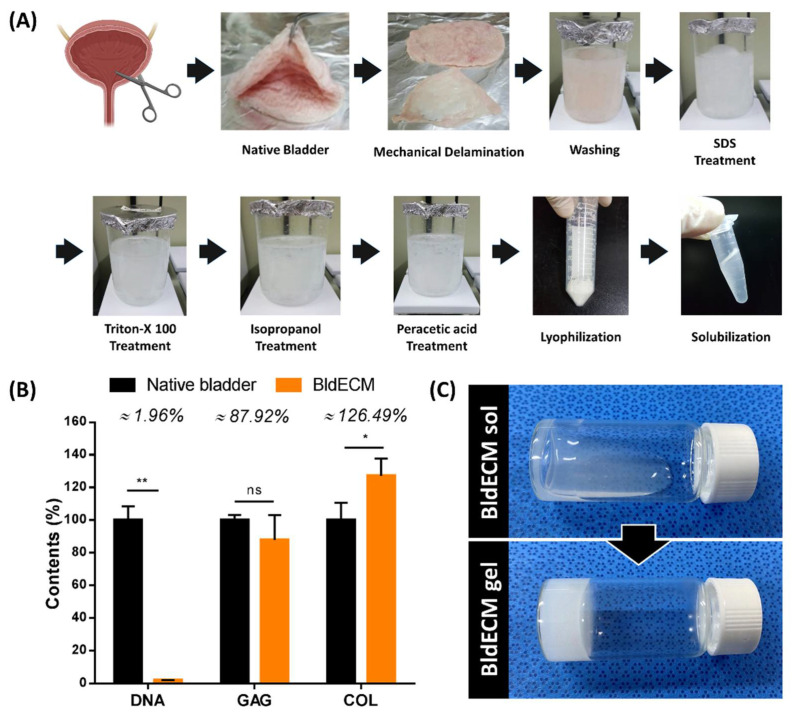
Preparation of the decellularized extracellular matrix from the bladder tissue. (**A**) Decellularization process through a series of physical, chemical, and enzymatic treatments. (**B**) Biochemical assays to evaluate decellularization, such as residual DNA, GAGs, and collagen contents in the BldECM and native bladder tissues (* *p* < 0.05 and ** *p* < 0.01; ns: Not significant). (**C**) Sol to gel transition of pH-adjusted BldECM hydrogel after incubation at 37 °C for 30 min.

Natural ECM is an intricate and heterogeneous network comprising various proteins, growth factors, and other molecules. Each tissue/organ has its own unique structure and composition [28] and replicating the complexity of native ECM properties can significantly benefit cellular behavior and its corresponding tissue/organ functions [18]. In this context, tissue/organ-derived dECMs are considered the most promising biomimetic material to achieve the desired tissue/organ-specificity, both structurally and biologically. In 3D bioprinting, using dECM bioinks can significantly help the production of highly biomimetic tissue constructs by supporting the complex interaction between cells and the microenvironments within the bioprinted tissues [18,29]. In this study, we extracted BldECM from porcine bladder tissue through decellularization using a series of physical, chemical, and enzymatic treatments (Figure 2A). To avoid immunological issues, cellular residues in the decellularized matrix were required to be less than 50 ng dsDNA/mg dry tissue [30]. Furthermore, it is necessary to preserve the major ECM components to recreate the tissue-specific microenvironments and modulate physiological cellular activities, such as adhesion, migration, proliferation, and differentiation. To evaluate the effectiveness of decellularization, we performed residual DNA quantification after decellularization of the bladder. Results showed that the DNA content was significantly reduced by 1.96% (40.25 ng/mg), while the GAG and collagen contents were retained by 87.92% and 126.49%, respectively (Figure 2B). Considering most cellular substances in the bladder were successfully removed after decellularization, we assume the overall compositional ratio in the dECM had changed, thereby proportionately increasing the collagen concentration in the decellularized tissue to over 100%. The results corroborated that the BldECM could successfully retain the extracellular compounds, and hence, can be conveniently used in bladder tissue engineering applications. The lyophilized BldECM was solubilized to create hydrogel for 3D bioprinting. The pre-gel solution of BldECM exhibited thermo-responsive behavior and transformed into a gel-like state under physiological conditions (pH = 7.4 and temperature 37 °C) (Figure 2C), which is a crucial characteristic of dECM bioinks for 3D bioprinting process [31]. The intramolecular binding of the solubilized dECM proteins in physiological pH and temperature conditions can support the mechanical stability of tissue constructs after 3D bioprinting [18,32]. Finally, the developed BldECM hydrogel was ready for cell encapsulation and 3D bioprinting applications.

### 3.2. Fabrication of a Bladder Mimicry Platform

**Figure 3:**
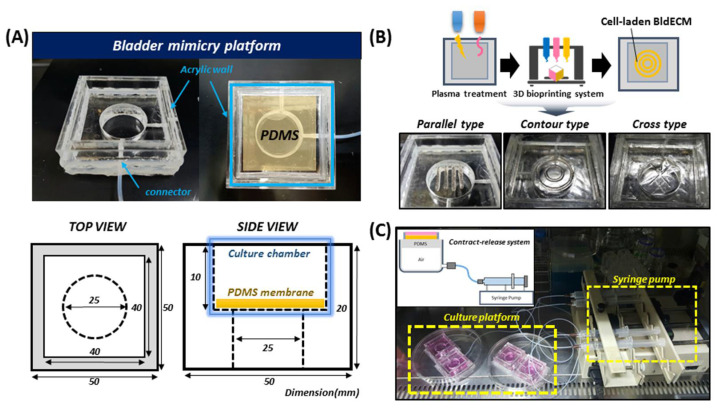
Development of a bladder mimicry platform that incorporates a contract-release system (CRS). (**A**) Design of the bladder mimicry platform comprising a vacuum chamber, medium container, and PDMS substrate; a snapshot of the bladder mimicry platform (above) and a detailed scheme of platform design (below, all dimensions are mm.) (**B**) 3D bioprinting of the bladder tissue models with various structures. Constructs of the parallel type (left), contour type (middle), and cross type (right). (**C**) Image of integrating a pressure-driven CRS into the model; cell-printed bladder tissues in the culture platform were stimulated mechanically by CRS for 10 days.

3D in vitro tissue models that replicate the microstructures and functions of real tissues/organs have emerged as predictive tissue modeling tools and a promising alternative to conventional animal testing [7,9,10]. In the present study, we devised a bladder mimicry platform inspired by the contractile motion of the native bladder smooth muscle (Figure 3). In a healthy urinary bladder, the mechanical tension generated by the smooth muscle elements significantly affects bladder compliance, which includes maintenance of contractility and elasticity [33,34]. The bioinspired device was developed by adapting a design concept based on the local smooth muscle contraction in the bladder wall. The device comprised an acrylic wall template (medium container), a PDMS membrane, and an acrylic baseplate (vacuum chamber). Using microfabrication approaches, the bladder-like device was reconstructed by assembling a thin PDMS membrane on the acrylic-based housing platform (Figure 3A). Furthermore, to mimic the physiological movement of the bladder wall, the elastic PDMS membrane was integrated into a housing platform and it was sandwiched between two acrylic frames. The top part of the wall template served to store culture medium, whereas the bottom part of the baseplate acted as a vacuum chamber enabling airflow.

Owing to the development of 3D bioprinting technology, it is possible to recreate biomimetic microenvironments with miscellaneous 3D structures using multiple biomaterials and cell types [18]. Accordingly, a bladder mimicry model was successfully fabricated by introducing cell-containing biomimetic bioink into the membrane substrate of the platform. The 3D bioprinting technique combined with the dECM bioink allowed in vitro fabrication of various structures with different shapes in an automated manner (Figure 3B and Video S1). Various types of bioprinted constructs were successfully fabricated by precisely depositing BldECM bioinks according to the pre-designed patterns, indicating the printability and fidelity of the developed BldECM bioink. This suggests that it is possible to directly produce the biological tissue constructs of desired shapes using dECM bioink and 3D bioprinting technology. In these models, bladder-specific environments, including ECM components, signaling molecules, and other junction proteins were reconstituted owing to the presence of bladder-derived matrices (BldECM). Furthermore, integrating a pressure-driven stimulation system into the model could reflect the moving nature of the cyclic and periodic behaviors of the bladder (Figure 3C). When air pressure is applied to the vacuum chamber, it exerts elastic deformation of the PDMS membrane, and the platform design replicates the physiology of urination activity controlled by the detrusor muscle in the elastic bladder wall. Therefore, it is amenable to mechanical manipulations into bioengineered tissue. Considering this, we developed a 3D bioprinting-based in vitro bladder model to recreate structural and mechanical features similar to an actual urinary bladder at a physiological level, which in turn can help develop bladder tissue in vitro and investigate its functionality.

### 3.3. Evaluation of the Biofunctionality of the Stem-Cell Laden Bladder dECM Bioink

**Figure 4:**
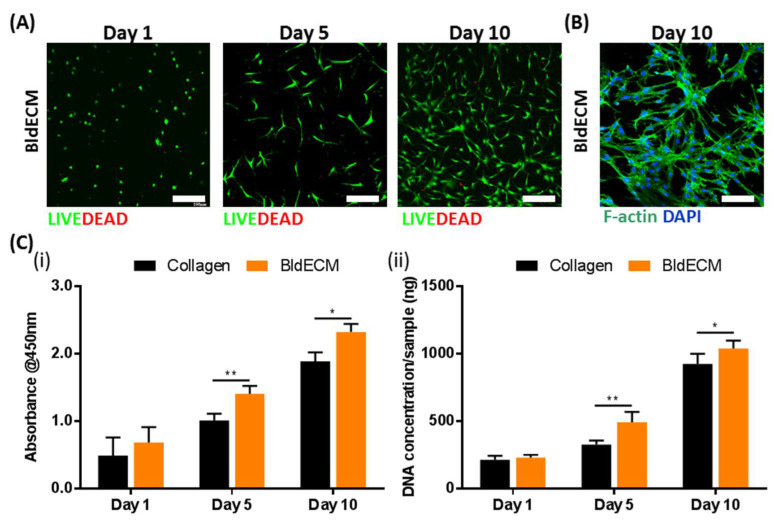
In vitro biofunctionality assessment of the hBMSCs-laden BldECM bioink. (**A**) Live/dead staining of hBMSCs in the BldECM for 10 days (scale bar = 200 μm); live cells (green), dead cells (red). (**B**) F-actin staining of the BldECM constructs on day 10 (scale bar = 100 μm); DAPI (blue, cell nuclei), F-actin (green, actin filaments). (**C**) Comparison of cell proliferation in collagen and BldECM groups for 10 days: (**i**) CCK-8 assay test and (**ii**) DNA quantification test (* *p* < 0.05 and ** *p* < 0.01).

For in vitro cell and tissue culture, cellular activities, such as viability, adhesion, and growth, are essential for investigating the biological properties of the biomaterials [35]. For the cell viability test, the live/dead images of hBMSCs in BldECM were taken on days 1, 5, and 10 (Figure 4A). Results showed that most cells were viable during the entire culture period and the values for cell viability were over 90% at every time point, thereby indicating that the BldECM material is biocompatible and has no cytotoxic effect on the encapsulated cells. By F-actin staining of the hBMSCs-laden BldECM bioink on day 10, we observed an elongated phenotype with interconnected networks within the BldECM matrix, which indicated excellent cell adhesion function of BldECM (Figure 4B). Considering the cell metabolic activity, CCK-8 assay and DNA quantification analysis were performed to evaluate the ability of the BldECM bioink to promote cell proliferation compared to the collagen bioink. CCK-8 test results showed that the absorbance values of all groups increased with time. Furthermore, the proliferation of the cells in the BldECM group was significantly higher than that in the collagen group on days 5 and 10 (Figure 4C(i)). A similar trend was observed in the DNA quantification analysis (Figure 4C(ii)). These results indicate that the BldECM bioink significantly contributed to the promotion of cell proliferation compared to the single component-based bioink (i.e., collagen). In conclusion, the BldECM bioink can provide a favorable physiological microenvironment for cell growth.

### 3.4. Effect of Physiological Stimuli on MSC Myogenesis in the Engineered In Vitro Bladder Model

**Figure 5:**
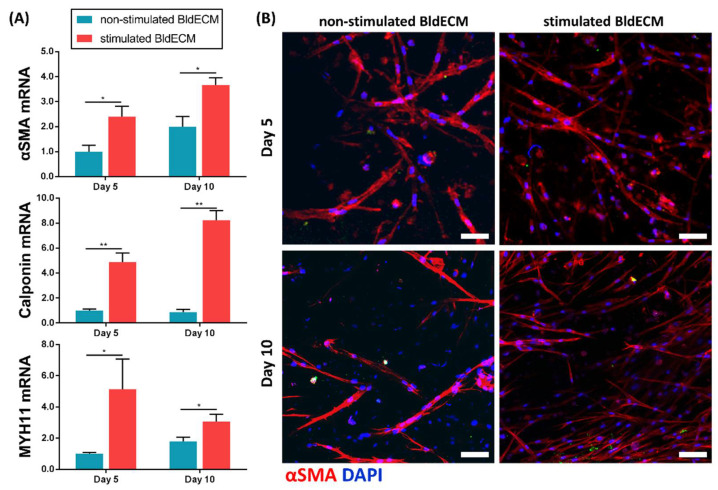
Effect of physiological stimuli on myogenesis in the engineered bladder model. (**A**) Changes in mRNA expression levels of myogenic markers (α-SMA, calponin, and MYH11) in the non-stimulated and stimulated BldECM groups at days 5 and 10 (* *p* < 0.05 and ** *p* < 0.01), and (**B**) Immunofluorescence staining of alpha-smooth muscle actin (αSMA) in the non-stimulated and stimulated BldECM groups on days 5 and 10 (scale bar = 100 μm); αSMA (red), DAPI (blue).

During normal urination in a healthy urination system, the urinary bladder experiences continuous changes in volume and surface area, while the detrusor muscle plays a major role in regulating the periodic process of bladder filling and voiding [34,36]. In this study, we replicated the coordinated motions of contraction and relaxation in the bladder by incorporating an air-flow control system into the platform design using a syringe pump apparatus (Video S2). When the air was injected into the chamber below the substrate, the PDMS membrane and the adherent cell-laden tissue constructs expanded. In contrast, when the air was eliminated from the device, the PDMS and the attached cellular constructs recovered their initial state. More importantly, external mechanical stimuli on the cell culture system supposedly promote cellular activities, such as growth, orientation, and differentiation, which are significant to engineer bladder tissue models [26,27]. We hypothesized that bioprinted constructs exposed to a particular mechanical stretching environment improve stem cell differentiation potential towards bladder smooth muscle lineage within the BldECM matrix. To test this hypothesis, we investigated the effect of physiologic mechanical stimulation on the myogenic differentiation of hBMSCs within the BldECM-derived in vitro model. By utilizing CRS, the bioprinted BldECM constructs underwent cyclic contraction-relaxation stretching for 10 d (stimulated BldECM group) and the representative myogenic markers were analyzed using RT-PCR tests and IF staining. The bioprinted constructs without mechanical stimulation were regarded as the control (non-stimulated BldECM group). In the experiment, we provided the constructs with cyclic stretching to mimic the expansion and relaxation movements of the bladder within a physiological range in a periodic manner. Results showed that all constructs exhibited elevated mRNA expressions of α-SMA, calponin, and MYH11 over 10 d of culture. Compared to the non-stimulated BldECM group at all experimental time points, hBMSCs in the stimulated BldECM group exhibited evident upregulation of myogenic markers (α-SMA, calponin, and MYH11), which demonstrated the conducive role of dynamic stretching on hBMSC myogenic lineage commitment (Figure 5A). To confirm the RT-PCR results, the production of the myogenic marker (αSMA) at a protein level was detected by IF staining over 10 d of culture. Corresponding to gene expression analyses, higher amounts of human-specific αSMA depositions produced by encapsulated hBMSCs in the constructs were found in the stimulated BldECM group compared to the control group on days 5 and 10 (Figure 5B). Moreover, in response to the mechanical stimulation, the cells in the constructs appeared to be aligned and elongated along the longitudinal construct axis, whereas random orientation was observed in the constructs without stimulation. These results indicated that the cell-laden BldECM constructs under physiological stretching could better enhance the myogenic differentiation of the hBMSCs.

To the best of our knowledge, this study is the first to combine BldECM bioink and a 3D bioprinted tissue model with a dynamic stimulation system to engineer bladder smooth muscle in vitro. Owing to their tissue-specific nature, tissue-specific dECMs significantly contribute to directing tissue-relevant MSC differentiation behavior and function [18,37]. Therefore, using BldECM can be highly benefitial in bladder tissue modeling approaches because most functional and structural components of the native ECM (e.g., many of the bladder ECM components, growth factors, and other bioactive signaling molecules) are preserved in BldECM. Considering the dynamic stimulation system, various types of mechanical cues are usually included as bioreactors specifically designed to emulate in vivo-like conditions, resulting in in vitro tissue maturation and functional improvements [38]. We aimed to simulate the in vivo dynamic mechanical stimulation to modulate cellular behaviors. Herein, adding dynamic mechanical cues in the bladder model considerably improved the MSC lineage specification into myogenic lineages, which demonstrated the importance of a biomechanical environment for tissue development and remodeling. Overall, our approach demonstrated the possibility of engineering a bladder mimicry platform that reconstructs physiological stretching for bladder tissue development in vitro. Moreover, this proof-of-concept system will lay the foundation for further research on systematic bladder disease modeling and dynamics of drug responses at cellular and tissue levels, thereby narrowing the gap between traditional cell culture systems and preclinical in vivo models.

## 4. Conclusions

The urinary bladder smooth muscle is highly responsible for maintaining the bladder contraction and relaxation movements of the bladder, which is essential for the regulation of urination. In this study, biomimetic tissue platform, tissue-specific dECM bioink, and mechanical stimulation were key elements to develop a bladder tissue model in vitro. Our results showed that the developed system improved the biofunctionality of the 3D bladder tissue in vitro. Our study also presents a proof-of-concept for the development of a bladder mimicry platform with a physiological mechanical stimulation system. It would be feasible to develop an effective bladder model with more complex functionalities when combining more relevant cell sources as well as biochemical and biophysical factors. Furthermore, the innovative features of the suggested in vitro model system will be of immense interest for the creation of dynamic in vitro models of other types of tissues/organs for various biomedical applications. Therefore, a bladder mimicry platform with CRS can help adopt 3D bioprinting-based biofabrication and develop dECM-derived complex tissue constructs for envisioned application in bladder disease modeling and drug-testing platforms.

## Figures and Tables

**Table 1:** Summary of the primary sequence used for the RT-PCR test.

Primer		Sequence
GAPDH	Forward	5′ CCTGGAGAAACCTGCCAAGTAT 3′
	Reverse	5′ CTCGGCCGCCTGCTT 3′
αSMA ^1^	Forward	5′ TGCCTGATGGGCAAGTGAT 3′
	Reverse	5′ TCTCTGGGCAGCGGAAAC 3′
Calponin	Forward	5′ GCCCGCCCACAACCA 3′
	Reverse	5′ GTGGCCCTAGGCGGAATT 3′
MYH11 ^2^	Forward	5′ ACAACCTGAGGGAGCGGTACT 3′
	Reverse	5′ CACGCAGAAGAGGCCAGAGT 3′

^1^ αSMA denotes α-smooth muscle actin; ^2^ MYH11 denotes smooth muscle myosin heavy chain 11.

## Supplementary Materials

The following are available online at https://www.mdpi.com/article/10.3390/mi13020277/s1, Video S1: 3D bioprinting of various bladder tissue units, Video S2: Bladder mimicry platform with CRS for mechanical stimulation.
